# Integrated Analysis Identifies DPP7 as a Prognostic Biomarker in Colorectal Cancer

**DOI:** 10.3390/cancers15153954

**Published:** 2023-08-03

**Authors:** Wei Zhang, Haidong Wang, Huadi Wang, Chuchu Xu, Rongjie Zhao, Junlin Yao, Chongya Zhai, Weidong Han, Hongming Pan, Jin Sheng

**Affiliations:** 1 Department of Medical Oncology, Sir Run Run Shaw Hospital, School of Medicine, Zhejiang University, Hangzhou 310016, China; 0618105@zju.edu.cn (W.Z.); haidongwang@zju.edu.cn (H.W.); 11518379@zju.edu.cn (H.W.); rogerzhao@zju.edu.cn (R.Z.); 11418280@zju.edu.cn (J.Y.); yu1988410@sina.com (C.Z.); hanwd@zju.edu.cn (W.H.); panhongming@zju.edu.cn (H.P.); 2 Department of Gastrointestinal Surgery, Shaoxing People’s Hospital (Shaoxing Hospital, Zhejiang University School of Medicine), Shaoxing 312000, China; chuchuxu@zju.edu.cn

**Keywords:** DPP7, colorectal cancer, cancer prognostic biomarker

## Abstract

**Simple Summary:**

Colorectal cancer (CRC) is one of the most common cancers, which has a poor prognosis and is prone to recurrence and metastasis. DPP7 is a prolyl peptidase, which is an enzyme characterized by the feature of cleaving proline-containing peptides. We found that the expression level of DPP7 in CRC samples was significantly higher than that in adjacent non-tumor tissues by analyzing public colorectal cancer data and surgical specimens of CRC patients. Moreover, the increased expression of DPP7 is correlated with a higher stage of cancer and shorter overall survival, indicating the diagnostic value of DPP7 for CRC. Furthermore, functional annotations indicated that DPP7 is involved in neuroactive ligand–receptor interaction and olfactory transduction signaling. Our data demonstrated that DPP7 is a promising diagnostic and prognostic biomarker as well as a new therapeutic target for CRC.

**Abstract:**

Colorectal cancer has a poor prognosis and is prone to recurrence and metastasis. DPP7, a prolyl peptidase, is reported to regulate lymphocyte quiescence. However, the correlation of DPP7 with prognosis in CRC remains unclear. With publicly available cohorts, the Wilcoxon rank-sum test and logistic regression were employed to analyze the relationship between DPP7 expression and the clinicopathological features of CRC patients. Specific pathways of differentially expressed genes were determined through biofunctional analysis and gene set enrichment analysis (GSEA). qPCR and immunohistochemical staining were used to determine DPP7 expression levels in surgical specimens. The public dataset and analysis of the biospecimens of CRC patients revealed that DPP7, in the CRC samples, was expressed significantly higher than in non-tumor tissues. Moreover, increased DPP7 was significantly associated with a higher N stage, lymphatic invasion, and shorter overall survival. Functionally, DPP7 is involved in neuroactive ligand–receptor interaction and olfactory transduction signaling. We identified a series of targeted drugs and small-molecule drugs with responses to DPP7. To conclude, DPP7 is a valuable diagnostic and prognostic biomarker for CRC and considered as a new therapeutic target.

## 1. Introduction

Colorectal cancer (CRC) remains the most common malignancy worldwide, and disease recurrence after surgery and metastasis are the major causes of the poor prognosis of CRC [1]. As highlighted in epidemiological studies, approximately 80% of CRC recurrence occurs within the first 3 years after primary tumor resection [2]. It is also instructive to note that the time interval from initial treatment to tumor recurrence is a vital prognostic factor for patients with recurrent CRC [3]. Therefore, there is an urgent need for the identification of novel prognostic biomarkers for CRC.

Prolyl peptidases are enzymes characterized by the feature of cleaving proline-containing peptides. Its members include dipeptidyl peptidase 4, DPP7 (also known as DPP2), DPP8, DPP9, prolyl endopeptidase, prolyl endopeptidase-like, and fibroblast activation protein [4]. DPP7 depicts a post-proline-cleaving aminopeptidase expressed in quiescent lymphocytes [5]. When stimulated by antigens, the lymphocytes may mobilize from their quiescent state to divide. The inhibition of DPP7 results in the apoptosis of resting but not activated lymphocytes [6]. These findings suggest that DPP7 is involved not only in the regulation of lymphocyte quiescence but also in immune regulation.

Accumulating evidence suggests that members of the DPP family of proteins are involved in the carcinogenesis and progression of various cancers [7,8]. Recently, an increasing number of studies have revealed the alteration of DPP7 in various cancers and that inhibiting DPP7 may efficiently increase the apoptosis of cancer cells [8,9]. A high expression of DPP7 is a poor prognostic factor for multiple myeloma patients [7]. Moreover, resistance to DPP7 inhibition-induced apoptosis was correlated with a more aggressive feature in chronic lymphocytic leukemia [8]. In addition, DPP7 has been identified as an important regulator of quiescence in lymphocytes [5]. Although DPP7 has been reported to be associated with disease and cancer [10,11], the prognostic value and the potential function of DPP7 in CRC and the connection between DPP7 and the immune landscape remain unclear.

In our study, original files were downloaded from the TCGA and GEO databases to analyze the relationship between DPP7 expression and clinicopathological features in CRC. We further constructed a predictive and prognostic model based on DPP7 expression level for patients with CRC. In addition, the DPP7-interacting protein network, DPP7-related pathways and biological functions were explored. To further investigate the correlation of DPP7 and the clinicopathological features of CRC patients, we used qPCR and immunohistochemical staining to analyze the expression level of DPP7 in surgical specimens. Consistent with the results from the bioinformatic analysis, the level of DPP7 in colorectal tumors was significantly higher than that in adjacent non-tumor tissues. Our findings may help to improve the early diagnosis rate of CRC, establishing a novel prognostic model for patients with CRC.

## 2. Materials and Methods

### 2.1. Data Acquisition and Genetic Alteration Analysis

The clinical–pathological information and gene expression data of CRC cases were collected from the TCGA COAD and READ databases. The clinical–pathological features of the two databases can be found in Table S1. The independent gene microarray data of GSE44076 from the Gene Expression Omnibus public database were applied as a validation cohort. Samples without corresponding clinical data were excluded from further analysis. According to the median value of DPP7 mRNA expression, the samples with DPP7 levels above the median were assigned to the high-expression group, and patients with DPP7 expression below the median were assigned to the low-expression group. The mRNA level of DPP7 (RNA Seq V2 RSEM) was analyzed using the cBioPortal database [12]. UALCAN were used to explore the DPP7 expression in colon cancer through the Clinical Proteomic Cancer Analysis Consortium dataset [13].

### 2.2. The Genetic Alteration of DPP7 and the Clinical Roles of DPP7 in CRC

All statistical analyses and plots in this study were performed and created using R software (Version 3.6.3). The expression of DPP7 in the unpaired and paired samples was analyzed using Wilcoxon’s rank-sum test and Wilcoxon signed-rank test, respectively. The visualization was presented with the “ggplot2” R packages (Version 3.4.2, https://cran.r-project.org/web/packages/ggplot2/index.html, accessed on 30 June 2023). The ROC curve was generated to evaluate the diagnostic ability of DPP7 using the pROC package (Version 1.18.4, https://cran.r-project.org/web/packages/pROC/index.html, accessed on 30 June 2023). Logistic regression was used to assess the relationship between clinicopathological features and DPP7 expression, and univariate and multivariate Cox regression analyses were applied to assess the prognostic role of DPP7 in patients with CRC. Bonferroni correction was used for multiple comparisons. The survival curve was generated using the Kaplan–Meier method with prognostic data obtained from previous research [14]. A nomogram based on the results of the multivariate analysis with the rms package (Version 6.7-0, https://cran.r-project.org/web/packages/rms/index.html, accessed on 30 June 2023) was constructed to individualize the prediction of 1-, 3-, and 5-year survival.

### 2.3. Biological Functional Analysis and Gene Set Enrichment Analysis (GSEA)

The gene expression data for the CRC cases were collected from the TCGA COAD and READ databases. Expression profile data (HTSeq-Counts) between the high-DPP7-expression group and the low-DPP7-expression group were analyzed and compared, and differentially expressed genes (DEGs) were further identified using the Wilcoxon rank-sum test. The threshold criteria were set as differences with a |log fold change| > 1.5 and adjusted *p*-value < 0.05. Each analysis step was repeated 1000 times for the reliability of the results. For functional or pathway items, adjusted *p*-values < 0.05 and a false discovery rate (FDR) threshold of 0.25 were used to identify significant differences [15]. The protein–protein interaction (PPI) network of DPP7 co-expressed genes was predicted based on the STRING (http://string-db.org; version 10.0) platform, and the interactions between proteins were also analyzed [16]. The main parameters were set as follows: the minimum required interaction score (“Low confidence (0.150)”), meaning of network edges (“evidence”), and size cutoff (“no more than 20 interactors”). Finally, the available experimentally determined DPP7-binding proteins were obtained, and gene ontology (GO) and Kyoto Encyclopedia of Genes and Genomes (KEGG) analysis was carried out to present the possible biological functions and signaling pathways affected by DPP7 [17]. GSEA [18] was performed using the R-packet ClusterProfiler [19] to demonstrate the significant functional and pathway differences between the DPP7 high-expression and low-expression groups.

### 2.4. Correlation between DPP7 and Drug Response

We collected the IC_50_ of 261 small molecules in 860 cell lines from Genomics of Drug Sensitivity in Cancer (GDSC, https://www.cancerrxgene.org/) and 481 small molecules in 1001 cell lines from the Genomics of Therapeutics Response Portal (CTRP, https://portals.broadinstitute.org/ctrp.v2.1/?page=#ctd2BodyHome), as well as the corresponding mRNA gene expression data, accessed on 30 June 2023. The mRNA expression data and drug sensitivity data were merged. To determine the strength of the association between gene expression and drug sensitivity, we used Pearson correlation analysis, adjusted for FDR.

### 2.5. RNA Extraction and Quantitative Real-Time RT-PCR

Thirty specimens of colorectal tumors and thirty corresponding normal tissues taken 10 cm from the tumors were obtained from Sir Run Run Shaw Hospital. This study was approved by the Ethics Committee of Sir Run Run Shaw Hospital (approval number: 20230629-023). Total RNA was extracted from the tissues with RNA Isolater Total RNA Extraction Reagent combined with chloroform, isopropanol, and 75% ethanol. cDNA synthesis was performed with the HiScript Ⅱ 1st Strand cDNA Synthesis Kit (Vazyme, R212-01). A CFX96 Touch Real-Time PCR Detection System (BIO-RAD) with MagicSYBR Mixture (Cwbio, CW3008M) was used to perform the qPCR analysis. The expression level of DPP7 was normalized to the GAPDH expression level, and 2^−∆Ct^ was used to exhaust the DPP7 mRNA expression level in the colorectal tumor and adjacent non-tumor tissues. The primers were as follows: for GAPDH, forward 5′-GGAGTCAACGGATTTGGTCGT-3′, reverse 5′-TCTCGCTCCTGGAAGATGGT-3′; and for DPP7, forward 5′-GAAGCGTTCCGACAGATCAAG-3′, reverse 5′-TCAGGTCCTTCTCGTCTGACA-3′.

### 2.6. Tissue Microarray and IHC

A tissue microarray of colorectal cancer (HColA180Su16) was purchased from Outdo Biotech (Shanghai, China). DPP7 antibody (NBP1-84986, Novusbio, Centennial, CO, USA) was used as the primary antibody. This study was approved by the Ethics Committee of Outdo Biotech (approval number: SHYJS-CP-1707004). From the tissue microarray of colorectal cancer, we obtained 99 colorectal tumor tissues to detect the expression level of DPP7, and the correlation between DPP7 expression and the clinicopathological features of CRC patients was analyzed. In total, 62 of the colorectal tumor tissues had the corresponding normal tissues (taken 10 cm from the tumor) and were used to compare the DPP7 expression level through a paired *t*-test. Immunohistochemical staining was scored according to the published methods [20]. Briefly, the staining scores are based on multiplying the staining intensity (no staining, 0; weak staining, 1; moderate staining, 2; strong staining, 3) and the percentage of positive cells (1–25%, 0; 26–50%, 1; 51–75%, 3; >75%, 4). The staining scores ranged from 0 to 12.

## 3. Results

### 3.1. The Expression of DPP7 in CRC Was Elevated

**Figure 1:**
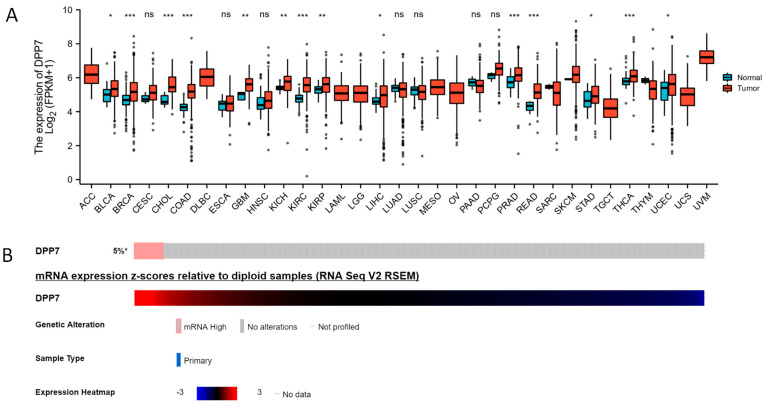
Increased expression of DPP7 in pan-cancer. (**A**) The expression of DPP7 in colorectal cancer and other cancers. DPP7 mRNA expression in the COAD, READ, and other types of human cancers from TCGA data. (**B**) Alteration of DPP7 in CRC based on the cBioPortal database with colorectal cancer samples. * *p* < 0.05, ** *p* < 0.01, and *** *p* < 0.001. ns refers to no significance.

**Figure 2:**
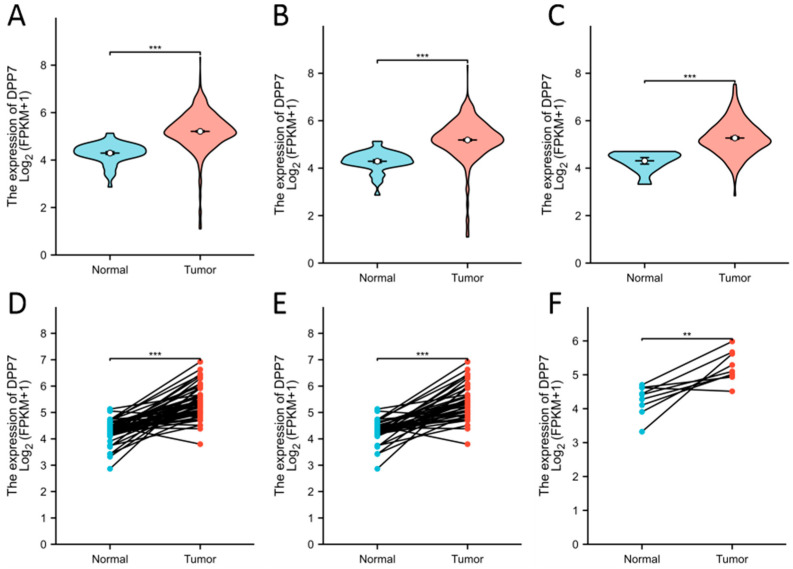
Elevated expression of DPP7 in CRC. (**A**) Expression of DPP7 in CRC (*n* = 619) and normal tissues (*n* = 51) in the COADREAD database. (**B**) Expression of DPP7 in colon cancer (*n* = 454) and normal tissues (*n* = 41) in the COAD database. (**C**) Expression of DPP7 in rectal cancer (*n* =165) and normal tissues (*n* = 10) in the READ database. (**D**) Expression of DPP7 in CRC (*n* = 50) and paired adjacent tissues. (**E**) Expression of DPP7 in colon cancer (*n* = 41) and paired adjacent tissues. (**F**) Expression of DPP7 in rectal cancer (*n* = 9) and paired adjacent tissues. ** *p* < 0.01, *** *p* < 0.001.

The expression analysis of DPP7 in pan-cancer and normal tissues indicated that the mRNA level of DPP7 was up-regulated in the colon adenocarcinoma (COAD) and rectum adenocarcinoma (READ) samples and other cancer types, including adrenocortical carcinoma (ACC), bladder urothelial carcinoma (BLCA), breast invasive carcinoma (BRCA), cervical squamous cell carcinoma and endocervical adenocarcinoma (CESC), cholangio carcinoma (CHOL), lymphoid neoplasm diffuse large B-cell lymphoma (DLBC), esophageal carcinoma (ESCA), glioblastoma multiforme (GBM), head and neck squamous cell carcinoma (HNSC), kidney chromophobe (KICH), kidney renal clear cell carcinoma (KIRC), kidney renal papillary cell carcinoma (KIRP), acute myeloid leukemia (LAML), brain lower grade glioma (LGG), liver hepatocellular carcinoma (LIHC), lung adenocarcinoma (LUAD), lung squamous cell carcinoma (LUSC), mesothelioma (MESO), ovarian serous cystadenocarcinoma (OV), pancreatic adenocarcinoma (PAAD), pheochromocytoma and paraganglioma (PCPG), prostate adenocarcinoma (PRAD), sarcoma (SARC), skin cutaneous melanoma (SKCM), stomach adenocarcinoma (STAD), testicular germ cell tumors (TGCT), thyroid carcinoma (THCA), thymoma (THYM), uterine corpus endometrial carcinoma (UCEC), uterine carcinosarcoma (UCS), and uveal melanoma (UVM) (Figure 1A). In addition, using the CbioPortal and TCGA database, a high mRNA expression of DPP7 was observed in 5% of the colon cancer samples (Figure 1B). The mRNA of DPP7 was highly expressed in the CRC tumor tissues (*n* = 619) compared with the normal tissues (*n* = 51, *p* < 0.001; Figure 2A–C). The expression of DPP7 at the mRNA level was significantly higher in the CRC tissues than that in the adjacent normal tissues (*p* < 0.001) (Figure 2D–F).

To confirm the conclusions from the TCGA dataset, we compared the expression of DPP7 in normal and CRC primary tissues based on GSE44076 dataset as external validation (Figure S1). As summarized in Table 1, high DPP7 mRNA expression was significantly associated with an advanced N stage (*p* = 0.004) and lymphatic invasion (*p* < 0.001). Further analysis revealed no statistically significant difference in DPP7 expression levels between the N0 and N1 groups (*p* = 0.49) or the N1 and N2 groups (*p* = 0.094). However, a significant difference in DPP7 expression levels was observed between the N0 and N2 groups (*p* = 0.010). Moreover, no significant associations with the other clinicopathologic features were revealed. These data suggest that DPP7 is overexpressed in CRC tissues, and higher DPP7 expression is closely related to CRC progression and metastasis.

### 3.2. Diagnostic and Prognostic Value of DPP7 Expression in CRC

**Figure 3:**
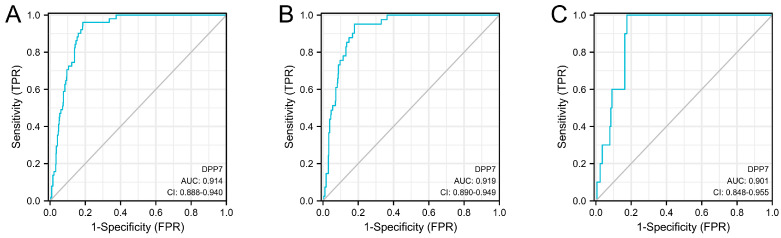
The diagnostic value of DPP7 in colorectal cancer patients. The AUC of DPP7 mRNA expression based on the COADREAD database (**A**), COAD database, (**B**) and READ database (**C**).

We first analyzed the diagnostic value of DPP7 in colorectal cancer patients. The AUC values of DPP7 in the COADREAD, COAD, and READ databases were 0.914 (95% CI: 0.888–0.940, Figure 3A), 0.919 (95% CI: 0.890–0.949, Figure 3B), and 0.901 (95% CI: 0.848–0.955, Figure 3C), respectively. This demonstrated that DPP7 is a potential diagnostic biomarker for CRC.

**Figure 4:**
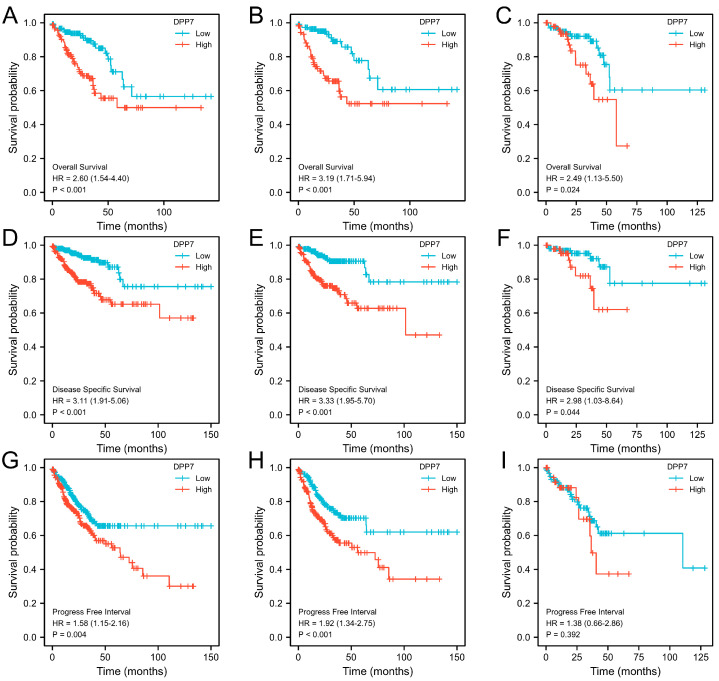
The relationship of DPP7 with survival in colorectal cancer patients in the TCGA database. OS in the COADREAD database (**A**), COAD database (**B**), and READ database (**C**); DSS in the COADREAD database (**D**), COAD database (**E**), and READ database (**F**); PFI in the COADREAD database (**G**), COAD database (**H**), and READ database (**I**). OS, overall survival; DSS, disease-specific survival; PFI, progress-free interval.

We further investigated the association between DPP7 expression levels and survival prognosis through Kaplan–Meier analyses. As shown in Figure 4, patients from the TCGA COADREAD dataset with high DPP7 expression levels a had shorter median OS (Figure 4A, HR = 2.60 95% CI, 1.54–4.40; *p* < 0.0.001), DSS (Figure 4D, HR = 3.11; 95% CI, 1.91–5.06; *p* < 0.001), and PFI (Figure 4G, HR = 1.58; 95% CI, 1.15–2.16; *p* = 0.004). The subgroup analyses of colon or rectal cancer also confirmed that patients with high DPP7 expression had a worse prognosis.

**Figure 5:**
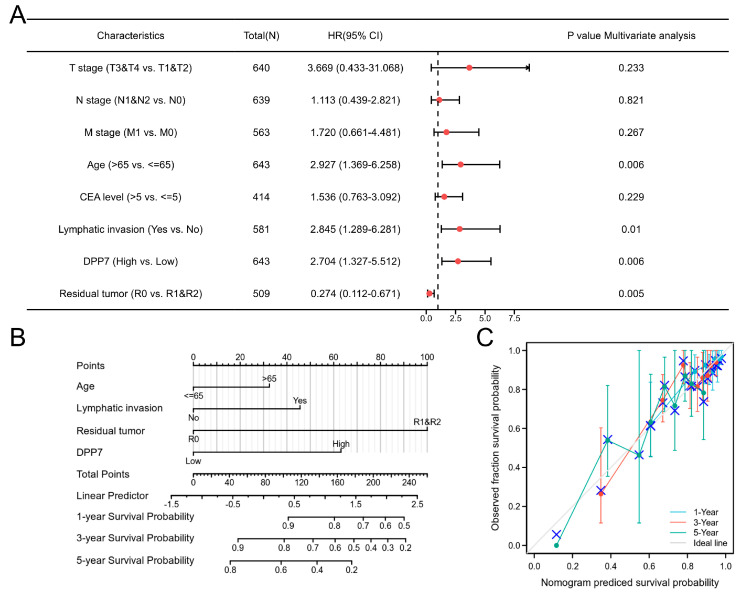
The prognostic role of DPP7 in CRC patients. (**A**) Multivariate regression analysis identified high DPP7 mRNA expression as an independent prognostic factor for OS in CRC patients. (**B**) Red rhombus represents hazard ratio. Nomogram for predicting the probability of 1-, 3-, and 5-year OS for CRC patients. (**C**) The C-index of the nomogram was 0.707 with 1000 bootstrap replicates (95% confidence interval: 0.673–0.742). Calibration plot of the nomogram for predicting OS likelihood.

Moreover, we identified the factors significantly associated with survival outcomes through univariate and multivariate Cox regression analyses. As shown in Table 2, an advanced T stage, N stage, individuals older than 65 years, a high level of CEA (>5 ng/mL), residual tumor or metastatic disease, lymphatic invasion, and high DPP7 expression were significantly associated with worse OS in the univariate analysis. Furthermore, the multivariate analysis (Figure 5A) confirmed the independent prognostic role of DPP7 in CRC (HR for OS: 2.704, 95%CI: 1.327–5.512, *p* = 0.006), along with age (HR for OS: 2.927, 95% CI: 1.369–6.258, *p* = 0.006), lymphatic invasion (HR for OS: 2.845, 95% CI: 1.289–6.281, *p* = 0.010), and status without residual tumor (HR for OS: 0.274, 95% CI: 0.112–0.671, *p* = 0.005). Taken together, these data indicated that DPP7 expression is an independent risk factor for CRC patients.

### 3.3. Construction and Evaluation of a Predictive Nomogram with DPP7

To provide a method for quantitatively predicting the prognosis of CRC, we constructed a novel nomogram, integrating the aforementioned clinical characteristics independently associated with survival. A point scale was used to assign these variables to the nomogram based on multivariate Cox analysis (age, lymphatic invasion, residual tumor, and DPP7 expression; Figure 5B). The 1-year, 3-year, and 5-year survival probabilities for CRC patients were determined based on vertical lines from the total point axis down to the outcome axis. The C-index of the nomogram was 0.707 (95% confidence interval: 0.673–0.742) after 1000 bootstrap replicates. The bias-corrected line in the calibration plot was close to the ideal curve (i.e., the 45-degree line), indicating that there is good agreement between the predicted value and the observed value (Figure 5C). Our result suggested that this nomogram is superior to a single prognostic factor in predicting long-term survival in CRC patients.

### 3.4. The PPI Network and DPP7-Related Pathways and Biological Functions

**Figure 6:**
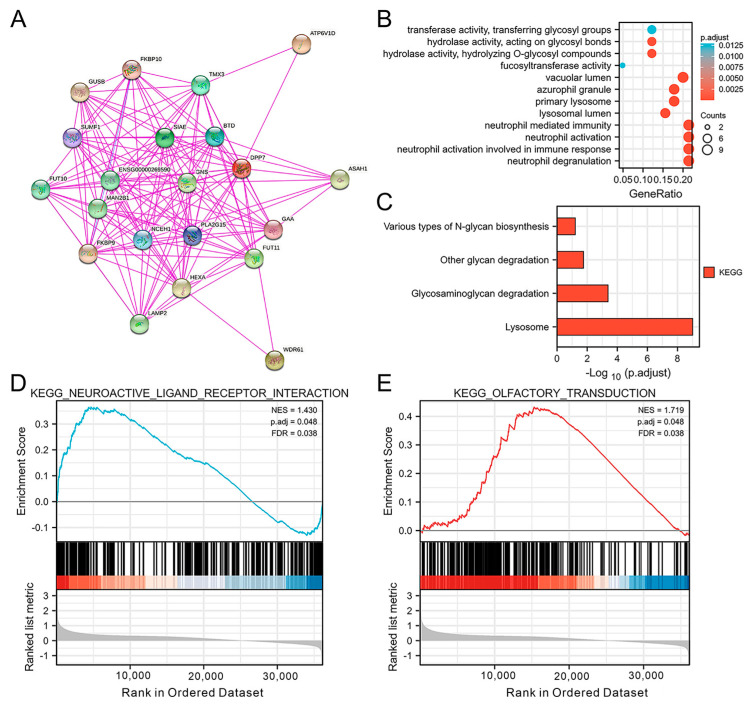
DPP7-associated protein–protein interaction network and pathway enrichment analysis. (**A**) GO enrichment plot of DPP7 in the CRC (**B**) KEGG pathway analysis based on DPP7 expression in the CRC (**C**) GSEA results, in turn based on the differentially expressed genes in high-DPP7-expression and low-DPP7-expression samples, identified significantly enriched pathways involving neuroactive ligand–receptor interaction (**D**) and olfactory transduction signaling (**E**). The count indicates the number of genes related to the enriched GO pathway. The color of the bar denotes the −log10 (p.adjust). NES, normalized enrichment score; FDR, false discovery rate.

Based on the STRING (https://string-db.org/, accessed on 30 June 2023) platform, a total of 20 DPP7-interacting proteins are presented. As shown in Figure 6A, the top five nodes with the highest degree centrality were sulfatase-modifying factor 1 (SUMF1), biotinidase (BTD), alpha glucosidase (GAA), sialic acid acetylesterase (SIAE), and fucosyltransferase 11 (FUT11). To understand the DPP7-related pathways and biological functions in CRC, we further analyzed the GO and KEGG pathways using the data obtained from the TCGA database. Cellular composition, molecular function, and biological processes were included in the GO enrichment analysis. Finally, we identified the top 12 enriched GO terms, as shown in Figure 6B. The top four enriched biological process terms were neutrophil degranulation, neutrophil activation, neutrophil activation involved in immune response, and neutrophil mediated immunity. The following cellular composition terms significantly correlated with DPP7 were vacuolar lumen, lysosomal lumen, primary lysosome, and azurophil granule. Moreover, the molecular function enrichment analysis showed that DPP7 was significantly correlated with hydrolase activity, acting on glycosyl bonds, hydrolyzing O-glycosyl compounds, fucosyltransferase activity, transferase activity, and transferring glycosyl groups. Interestingly, the KEGG pathway analysis demonstrated that DPP7 was involved in pathways related to the lysosome, glycosaminoglycan degradation, other glycan degradation, and various types of N-glycan biosynthesis (Figure 6C). These findings indicated that DPP7 may play a role in the regulation of neutrophil activation, glycan metabolism, and the function of lysosomes. The GSEA results based on the differentially expressed genes in the high-DPP7-expression and low-DPP7-expression samples identified significantly enriched pathways involving neuroactive ligand–receptor interaction (NES = 1.430; *p* = 0.048; FDR = 0.038, Figure 6D) and olfactory transduction signal (NES = 1.719; *p* = 0.048; FDR = 0.038, Figure 6E).

### 3.5. Correlation between DPP7 and Drug Response

**Figure 7:**
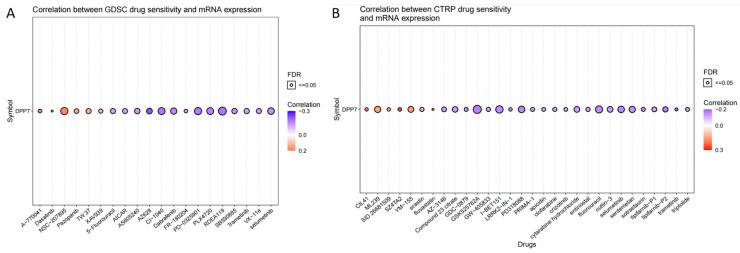
The correlation between DPP7 expression and drug sensitivity. (**A**) The correlation between sensitivity and DPP7 mRNA expression in the GDSE database (top 20). (**B**) The correlation between sensitivity and DPP7 mRNA expression in the CTRP database. (top 30). The gene set drug resistance analysis of the GDSC/CTRP IC50 drug data. Blue bubbles represent negative correlations; red bubbles represent positive correlations. The deeper of color is, the higher the correlation is. Bubble size is positively correlated with FDR significance. The black outline border indicates FDR ≤ 0.05. A positive correlation means that the gene with high expression is resistant to the drug, and vice versa.

To identify the correlation between DPP7 expression and predicted drug efficacy, 261 small molecules in 860 cell lines from GDSC and 481 small molecules in 1001 cell lines from CTRP were collected and analyzed with respect to the DPP7 mRNA expression level (Table S2). The top 30 drugs were ranked using the integrated level of correlation coefficient and FDR of the searched genes, which had to be >0.1 with an FDR < 0.05. We then multiplied the -log10FDR and the absolute value of the correlation coefficient to obtain a score for each drug–gene pair. The total score for each drug was obtained by adding up the scores for all the pairs associated with that drug. The resulting data are visualized in Figure 7, where the genes and drugs are ranked according to their scores. Positive correlations between DPP7 expression and drug sensitivity are represented by red bubbles, which means that the higher DPP7 expression is, the more resistant the gene is to the indicated drug. Negative correlations between DPP7 expression and drug sensitivity are denoted by blue bubbles, which means that the higher the DPP7 level is, the less resistant the gene is to the indicated drug. The level of correlation is indicated by the color intensity, with deeper colors signifying higher correlations. The size of the bubble is positively correlated with the FDR significance of the association. The black outline border in the plot indicates an FDR of ≤0.05.

As Figure 7 shows, the expression of DPP7 exhibited a negative correlation with the efficacy of several drugs, such as fluorouracil, dabrafenib, and selumetinib, frequently prescribed for colon cancer, providing a potential link between DPP7 expression and the sensitivity of cancer cells to various drugs.

### 3.6. DPP7 Is Highly Expressed in Patients with Colorectal Cancer and Associated with a Poor Prognosis

**Figure 8:**
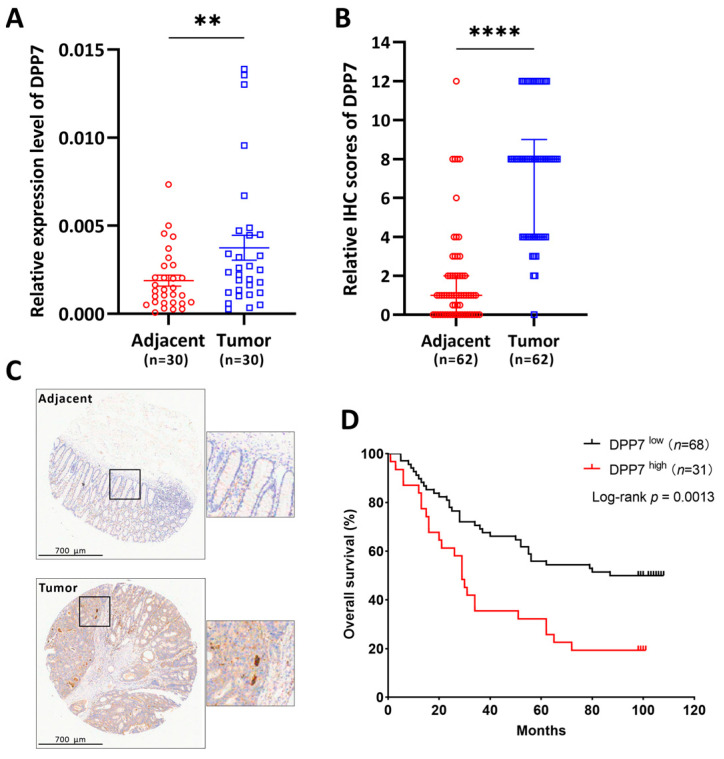
DPP7 expresses highly in colorectal cancer and predicts poor prognosis. (**A**) DPP7 mRNA expression in colorectal tumor and paired adjacent non-tumor tissues. (**B**) IHC examination of DPP7 protein expression in colorectal tumor and paired adjacent non-tumor tissues. (**C**) The representative IHC image of DPP7 in colorectal tumor and adjacent non-tumor biopsies. (**D**) Overall survival of CRC patients with high (IHC score = 12) and low (IHC score < 12) DPP7 protein expression. ** *p* < 0.01; **** *p* < 0.0001. The *p*-value was determined through a paired Student’s *t*-test (**A**,**B**); the survival curve was analyzed with a log-rank test (**D**).

To further investigate the expression level in human colorectal cancer, we used qPCR to detect the expression level of DPP7 in colorectal tumor tissues and their paired adjacent normal tissues at the mRNA level. As Figure 8A shows, DPP7 was significantly increased in the tumor compared with the adjacent non-tumor tissues. Next, we used a tissue microarray to determine DPP7 expression at the protein level and evaluate the clinical relevance of DPP7. We found that DPP7 was expressed higher in the tumor range than in the paired adjacent non-tumor tissues (Figure 8B,C), and the increased expression of DPP7 predicted shorter overall survival (Figure 8D). Therefore, DPP7 is aberrantly overexpressed in colorectal tumors and is closely associated with the poor prognosis of patients with colorectal cancer.

## 4. Discussion

DPP7 is known to be correlated with disease and cancer, but the prognostic value of DPP7 in CRC remains unclear. This study found that DPP7 expression in CRC samples was significantly higher than that in normal tissues by analyzing public CRC data and clinical bio-specimens. Furthermore, the enhanced expression level of DPP7 is correlated with high-grade tumors and low overall survival, indicating the diagnostic value of DPP7 for CRC.

In the present study, we first explored the prognostic value of DPP7 in the TCGA and validated our findings based another 98 CRC specimens and 148 normal tissues from the GSE44076 database. Both the mRNA and protein levels of DPP7 were significantly higher in the colon cancer samples than in the normal tissues. After verifying the differential expression of DPP7 in the colon cancer and adjacent tissues, we further analyzed the prognostic and diagnostic value of DPP7. Several studies have explored the prognostic value of DPP7 in cancers. Our results demonstrated that a high expression of DPP7 was associated with a poor prognosis, including a shorter OS, PFI, and DSS for patients with CRC. Our results are consistent with a previous study of CRC. Ahluwalia P et al. [21] established a high prognostic score, composed of four gene signatures including DPP7, which can predict a poor prognosis in CRC patients. However, a recent study found that a high expression level of DPP7 was associated with a good prognosis in breast cancer [10]. Comparatively, DPP7 may play different prognostic roles in different cancer types, and our study first clarified the prognostic value of DPP7 as a single gene in CRC. We further constructed a nomogram incorporating the expression level of DPP7 and another three clinicopathologic variables including age, lymphatic invasion, and residual tumor. The positions of these variables were accumulated and recorded as the total points. According to the nomogram, DPP7 expression contributed the most extreme data points (ranging from 0 to 100) compared with the other clinical variables within the nomogram, which was consistent with the results of the multivariate Cox regression.

To identify the potential relationships between DPP7 and other proteins in CRC, we conducted a PPI network analysis and identified the top five nodes with the highest degree centrality: sulfatase-modifying factor 1 (SUMF1), biotinidase (BTD), alpha glucosidase (GAA), sialic acid acetylesterase (SIAE), and fucosyltransferase 11 (FUT11). Recent studies have shown that most of the abovementioned genes are associated with the pathogenesis, progression, and prognosis of various cancers [22,23,24,25,26,27]. We further conducted enrichment analyses, and the results demonstrated that DPP7 was significantly correlated with fucosyltransferase activity. The previous work of Deschuyter M et al. proved that the overexpression of O-fucosyltransferase 1 in many cancers, including CRC, leads to NOTCH signaling dysregulation associated with carcinogenesis [28]. These results suggest that DPP7 may affect the expression of O-fucosyltransferase 1 in CRC by regulating the activity of fucosyltransferase and thereby alter the biological features of tumors. Furthermore, we integrated the potential biological functions of DPP7-expression-related genes with a series of enrichment analyses and identified the potential impacts of “neutrophil activation” and “neutrophil mediated immunity” on the etiology or pathogenesis of CRC. The role of DPP7 on neutrophil-associated immune responses has not been reported. However, DPP1, another member of the dipeptidyl peptidase family, activates neutrophil serine proteases to promote neutrophil maturation [29]. Inhibitors of DPP1, such as brensocatib, were used to treat bronchiectasis in several clinical trials [30,31,32]. There is increasing evidence to show that tumor-associated neutrophils (TANs) play an important role in cancer progression. Previous studies have extensively described TAN as a key driver of cancer progression, including cancer proliferation, aggressiveness, and dissemination, as well as immune suppression [33,34]. However, such studies only focused on the late stages of tumorigenesis, in which chronic inflammation is most prominent. The role of TANs in early tumor stages remains poorly understood. The question of whether DPP7 regulates TAN activation and immune regulation in the early tumor stages of CRC needs further study and verification.

The expression of DPP7 exhibited a negative correlation with the efficacy of several drugs, such as fluorouracil, dabrafenib, and selumetinib, frequently prescribed for colon cancer. In summary, our study generated valuable insights into the link between the expression of specific genes and the sensitivity of cancer cells to various drugs. The robust analytical approach used in this study has the potential to inform future drug development efforts by identifying novel drug targets and biomarkers that could enhance patient outcomes.

To the best of our knowledge, the present study was the first to identify DPP7 as a promising diagnostic biomarker and an independent prognostic factor for CRC. However, some limitations need to be considered. First, future studies need to validate the nomogram we proposed. Second, we need to carry out further studies on cell lines and animal models to elucidate the biological function and mechanism of DPP7 in colorectal cancer cells.

## 5. Conclusions

DPP7 may serve as a novel diagnostic and prognostic biomarker for CRC, probably via its potential role in fucosyltransferase activity. It is plausible that DPP7 may serve as a promising therapeutic target for CRC. The regulative mechanism of DPP7 in colorectal cancer cells deserves further investigation.

## Figures and Tables

**Table 1:** The relationships between DPP7 expression and clinicopathological features in CRC patients based on the TCGA database (*n* = 644).

Characteristic	Low Expression of DPP7	High Expression of DPP7	*p*
*n*	322	322	
Age, meidan (IQR)	68 (57, 75)	68 (58.25, 77)	0.210
Gender, *n* (%)			0.752
Female	148 (46.0%)	153 (47.5%)	
Male	174 (54.0%)	169 (52.5%)	
Race, *n* (%)			0.628
Asian	6 (2.8%)	6 (3.3%)	
Black or African American	41 (19.2%)	28 (15.6%)	
White	167 (78.0%)	146 (81.1%)	
Age, *n* (%)			0.474
≤65	143 (44.4%)	133 (41.3%)	
>65	179 (55.6%)	189 (58.7%)	
T stage, *n* (%)			0.377
T1	11 (3.4%)	9 (2.8%)	
T2	58 (18.2%)	53 (16.5%)	
T3	220 (69.0%)	216 (67.1%)	
T4	30 (9.4%)	44 (13.7%)	
N stage, *n* (%)			0.038
N0	195 (61.3%)	173 (53.7%)	
N1	76 (23.9%)	77 (23.9%)	
N2	47 (14.8%)	72 (22.4%)	
M stage, *n* (%)			0.138
M0	236 (86.8%)	239 (81.8%)	
M1	36 (13.2%)	53 (18.2%)	
Pathologic stage, *n* (%)			0.076
Stage I	57 (18.4%)	54 (17.3%)	
Stage II	131 (42.3%)	107 (34.2%)	
Stage III	86 (27.7%)	98 (31.3%)	
Stage IV	36 (11.6%)	54 (17.3%)	
CEA level, *n* (%)			0.830
≤5	133 (63.6%)	128 (62.1%)	
>5	76 (36.4%)	78 (37.9%)	
Residual tumor, *n* (%)			0.082
R0	228 (92.7%)	240 (90.9%)	
R1	5 (2.0%)	1 (0.4%)	
R2	13 (5.3%)	23 (8.7%)	
Perineural invasion, *n* (%)			0.977
No	91 (74.0%)	84 (75.0%)	
Yes	32 (26.0%)	28 (25.0%)	
Lymphatic invasion, *n* (%)			<0.001
No	198 (67.3%)	152 (52.8%)	
Yes	96 (32.7%)	136 (47.2%)	
Neoplasm type, *n* (%)			0.787
Colon adenocarcinoma	237 (73.6%)	241 (74.8%)	
Rectum adenocarcinoma	85 (26.4%)	81 (25.2%)	

**Table 2:** Univariate and multivariate Cox proportional hazards analysis of DPP7 expression and OS for patients with CRC based on the TCGA database.

Characteristics	Total (*n*)	Univariate Analysis		Multivariate Analysis	
		HR (95% CI)	*p*-Value	HR (95% CI)	*p*-Value
T stage (T3 and T4 vs. T1 and T2)	640	2.468 (1.327–4.589)	0.004	3.669 (0.433–31.068)	0.233
N stage (N1 and N2 vs. N0)	639	2.627 (1.831–3.769)	<0.001	1.113 (0.439–2.821)	0.821
M stage (M1 vs. M0)	563	3.989 (2.684–5.929)	<0.001	1.720 (0.661–4.481)	0.267
Gender (Male vs. Female)	643	1.054 (0.744–1.491)	0.769		
Age (>65 vs. ≤65)	643	1.939 (1.320–2.849)	<0.001	2.927 (1.369–6.258)	0.006
CEA level (>5 vs. ≤5)	414	2.620 (1.611–4.261)	<0.001	1.536 (0.763–3.092)	0.229
Perineural invasion (Yes vs. No)	235	1.692 (0.907–3.156)	0.099		
Lymphatic invasion (Yes vs. No)	581	2.144 (1.476–3.114)	<0.001	2.845 (1.289–6.281)	0.010
DPP7 (High vs. Low)	643	2.094 (1.462–2.998)	<0.001	2.704 (1.327–5.512)	0.006
Residual tumor (R0 vs. R1 and R2)	509	0.217 (0.132–0.357)	<0.001	0.274 (0.112–0.671)	0.005
Neoplasm type (rectum adenocarcinoma vs. colon adenocarcinoma)	643	0.799 (0.519–1.230)	0.308		
Race (White vs. Black or African American and Asian)	394	0.933 (0.541–1.607)	0.802		

Abbreviations: CI, confidence interval; HR, hazard ratio.

## Data Availability

Publicly available datasets were analyzed in this study. The data can be found here: The Cancer Genome Atlas (https://portal.gdc.cancer.gov/), accessed on 30 June 2023. Any additional information required can be made available upon reasonable request to the corresponding author.

## Supplementary Materials

The following supporting information can be downloaded at: https://www.mdpi.com/article/10.3390/cancers15153954/s1, Figure S1. The expression of DPP7 in normal and CRC primary tissues based on GSE44076 dataset. Table S1. The clinicopathological features in CRC patients based on the TCGA COAD and READ databases. Table S2. Correlation between mRNA expression of DPP7 and cancer drug sensitivity of GDSC and CTRP.
