# Supplementary material for: Integrated Analysis Identifies DPP7 as a Prognostic Biomarker in Colorectal Cancer
Source: Cancers (Basel). 2023 Aug 3;15(15):3954. doi: 10.3390/cancers15153954 (PMC10416901; doi:10.3390/cancers15153954)
Supplement: Supplementary file 1 [file cancers-15-03954-s001.zip › cancers-2516876-supplementary/Supplementary/Figure S1 and Table S1.pdf]

Figure S1

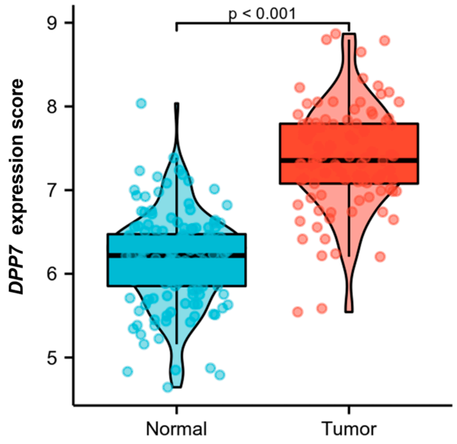

Figure S1. The expression of DPP7 in normal and CRC primary tissues based on GSE44076 dataset.

Table S1. The clinicopathological features in CRC patients based on the TCGA COAD and READ databases.

Characteristic	classification	COAD database	READ database
		cases	cases
n		478	166
Gender, n (%)	Female	226 (47.3%)	75 (45.2%)
	Male	252 (52.7%)	91 (54.8%)
Race, n (%)	Asian	11 (3.6%)	1 (1.1%)
	Black or African American	63 (20.6%)	6 (6.8%)
	White	232 (75.8%)	81 (92%)
Age, n (%)	<=65	194 (40.6%)	82 (49.4%)
	>65	284 (59.4%)	84 (50.6%)
T stage, n (%)	T1	11 (2.3%)	9 (5.5%)
	T2	83 (17.4%)	28 (17.1%)
	T3	323 (67.7%)	113 (68.9%)
	T4	60 (12.6%)	14 (8.5%)
N stage, n (%)	N0	284 (59.4%)	84 (51.9%)
	N1	108 (22.6%)	45 (27.8%)
	N2	86 (18%)	33 (20.4%)
M stage, n (%)	M0	349 (84.1%)	126 (84.6%)
	M1	66 (15.9%)	23 (15.4%)
Pathologic stage, n (%)	Stage I	81 (17.3%)	30 (19.2%)
	Stage II	187 (40%)	51 (32.7%)
	Stage III	133 (28.5%)	51 (32.7%)
	Stage IV	66 (14.1%)	24 (15.4%)
Primary therapy outcome, n (%)	PD	25 (10%)	8 (12.9%)

Characteristic	classification	COAD database	READ database
		cases	cases
	SD	4 (1.6%)	1 (1.6%)
	PR	13 (5.2%)	3 (4.8%)
	CR	208 (83.2%)	50 (80.6%)
BMI, n (%)	<25	87 (34%)	20 (27.4%)
	>=25	169 (66%)	53 (72.6%)
Residual tumor, n (%)	R0	346 (92.5%)	122 (89.7%)
	R1	4 (1.1%)	2 (1.5%)
	R2	24 (6.4%)	12 (8.8%)
CEA level, n (%)	<=5	196 (64.7%)	65 (58%)
	>5	107 (35.3%)	47 (42%)
Lymphatic invasion, n (%)	NO	266 (61.3%)	84 (56.8%)
	YES	168 (38.7%)	64 (43.2%)
Perineural invasion, n (%)	NO	135 (74.6%)	40 (74.1%)
	YES	46 (25.4%)	14 (25.9%)
